# Prospective evaluation of the performance of [^68^Ga]Ga-PSMA-11 PET/CT(MRI) for lymph node staging in patients undergoing superextended salvage lymph node dissection after radical prostatectomy

**DOI:** 10.1007/s00259-019-04361-0

**Published:** 2019-06-29

**Authors:** Mohammad Abufaraj, Bernhard Grubmüller, Markus Zeitlinger, Gero Kramer, Christian Seitz, Andrea Haitel, Pascal Baltzer, Marcus Hacker, Wolfgang Wadsak, Sarah Pfaff, Tomasz Wiatr, Markus Mitterhauser, Shahrokh F. Shariat, Markus Hartenbach

**Affiliations:** 1 Department of Urology, Medical University of Vienna, Vienna General Hospital, Währinger Gürtel 18-20, 1090 Vienna, Austria; 2 Division of Urology, Department of Special Surgery, Jordan University Hospital, The University of Jordan, Amman, Jordan; 3 Working Group of Diagnostic Imaging in Urology, Austrian Society of Urology, Vienna, Austria; 4 Department of Clinical Pharmacology, Medical University of Vienna, Vienna, Austria; 5 Ludwig Boltzmann Institute for Applied Diagnostics, Vienna, Austria; 6 Department of Pathology, Medical University of Vienna, Vienna, Austria; 7 Department of Biomedical Imaging and Image-guided Therapy, Division of General and Pediatric Radiology, Medical University of Vienna, Vienna, Austria; 8 Department of Biomedical Imaging and Image-guided Therapy, Division of Nuclear Medicine, Medical University of Vienna, Vienna, Austria; 9 Center for Biomarker Research in Medicine, CBmed GmbH, Graz, Austria; 10 Department of Urology, Jagiellonian University, Collegium Medicum, Cracow, Poland; 11 Karl Landsteiner Institute of Urology and Andrology, Vienna, Austria; 12 Department of Urology, University of Texas Southwestern Medical Center, Dallas, TX USA; 13 Department of Urology, Weill Cornell Medical College, New York, NY USA; 14 Institute for Urology and Reproductive Health, Sechenov University, Moscow, Russia

**Keywords:** Biochemical recurrence, Hybrid imaging, PET/CT, PET/MRI, PSMA ligand, Salvage lymph node dissection

## Abstract

**Purpose:**

To assess the accuracy of [^68^Ga]-PSMA-11 PET/CT or [^68^Ga]-PSMA-11 PET/MRI (PSMA-11 PET/CT(MRI)) for lymph node (LN) staging using salvage LN dissection (SLND) in patients with biochemical recurrence (BCR) after radical prostatectomy (RP).

**Patients and methods:**

In a prospective study, 65 consecutive patients who developed BCR after RP underwent SLND after PSMA-11 PET/CT(MRI) between 2014 and 2018. Extended SLND up to the inferior mesenteric artery was performed in all patients. Regional and template-based correlations between the presence of LN metastases on histopathology and whole-body PSMA-11 PET/CT(MRI) results were evaluated. The diagnostic accuracy of PSMA-11 PET/CT(MRI) was also evaluated in relation to PSA level at the time of SLND.

**Results:**

The median age of the patients at the time of SLND was 65 years (IQR 63–69 years) and the median PSA level was 1.4 ng/ml (IQR 0.8–2.9 ng/ml). Before SLND, 50 patients (77%) had additional therapy after RP (26.2% androgen-deprivation therapy and 50.8% radiotherapy). The median number of LNs removed on SLND was 40 (IQR 33–48) and the median number of positive nodes was 4 (IQR 2–6). LN metastases were seen in 13.8% of resected LNs (317 of 2,292). LNs positive on PSMA-11 PET/CT(MRI) had a median diameter of 7.2 mm (IQR 5.3–9 mm). Metastatic LNs in regions negative on PSMA-11 PET had a median diameter of 3.4 mm (IQR 2.1–5.4 mm). In a regional analysis, the sensitivity of PSMA-11 PET/CT(MRI) ranged from 72% to 100%, and the specificity from 96% to 100%. Region-specific positive and negative predictive values ranged from 95% to 100% and 93% to 100%, respectively.

**Conclusion:**

PSMA-11 PET/CT(MRI) has a very good performance for the identification of LN metastases in patients with BCR after RP. The high diagnostic accuracy in the regional and subregional analyses demonstrates the potential of this approach to enable a region-directed instead of a complete bilateral therapeutic intervention. The performance of PSMA-11 PET/CT(MRI) is dependent on the PSA level and the size of the metastatic deposit.

## Introduction

Despite effective local therapy with curative intent, about one-third of patients with localized prostate cancer (PC) experience biochemical recurrence (BCR) [1–3]. Rising prostate-specific antigen (PSA) in these patients may be due to locoregional recurrence, distant metastases, or both [2, 4, 5]. While patients with distant metastases often require systemic therapy, those with locoregional failure are candidates for targeted locoregional salvage therapies with or without a limited course of androgen-deprivation therapy (ADT) [2, 6]. Indeed, locoregional therapy such as salvage radiotherapy or lymphadenectomy has been shown to result in potentially durable local and distant cancer control [7]. Contemporary reports indicate that approximately one-third of patients treated with radical prostatectomy (RP) do not receive a pelvic lymph node dissection (PLND) [8]. The rate and extent of PLND as well as the number of lymph nodes (LNs) removed have continuously decreased over the last three decades. Consequently, the frequency of “limited” pelvic LN metastases as a cause of BCR is expected to increase [8].

Novel imaging techniques such as positron emission tomography (PET) with specific radiopharmaceuticals targeting prostate-specific membrane antigen (PSMA), that avidly accumulates in PC foci, have improved tumour staging [9] and have led to earlier detection and localization of specific metastases at the time of BCR [2, 10–12]. While choline PET has shown promising early results, the rates of false-positive and false-negative results limit its widespread use, especially in low-volume disease such relapse in single LNs [13]. Furthermore, the performance of choline PET/CT is lower in patients with low PSA levels, limiting its value for guiding “lesion-targeted” salvage lymph node dissection (SLND) [1, 14, 15]. In contrast, [^68^Ga]-PSMA-11 ([^68^Ga]Ga-PSMA^HBED-CC^; PSMA-11) has shown very promising diagnostic performance in patients with rising but still low PSA levels after primary local therapy with curative intent such as RP [12, 16, 17]. Indeed, PSMA-11 PET imaging has shown very good results in patients with LN metastasis [17].

We therefore hypothesized that PSMA-11 PET imaging could help reliably identify patients who harbour LN-only metastases after RP and help localize them with sufficient accuracy to allow local therapy. Given the currently unmet need to accurately identify isolated LN metastases in patients with BCR after RP, we performed a prospective study to assess the performance of PSMA-11 PET/CT or PSMA-11 PET/MRI (PSMA-11 PET/CT(MRI)) prior to SLND. Our primary endpoint was to assess the concordance between PSMA-positive LNs and histopathology as the confirmatory test. Our second aim was to determine the size of LN metastasis needed to obtain a reliably positive PSMA-11 PET signal.

## Materials and methods

### Patients

We performed a prospective analysis in the scope of an ongoing clinical trial (ClinicalTrials.gov NCT02974075, clinicaltrials.gov) in patients with BCR following RP from May 2014 to February 2018. A total of 65 consecutive patients had LN-only metastasis on PSMA-11 PET/CT(MRI) and agreed to undergo an open SLND. BCR was defined as two consecutive increases in PSA level above 0.2 ng/ml. None of the patients had evidence of local recurrence, or visceral or bone metastasis at the time of SLND. All resected LNs were formalin-fixed, paraffin-embedded and processed according to the American Joint Committee on Cancer TNM staging system and WHO/ISUP 2005 system by a dedicated urohistopathologist who was blinded to the imaging results [18]. PSMA-11 PET/CT(MRI) without additional imaging was performed to assess the site of BCR after SLND. Follow-up schedules included physical examination and PSA measurement every 3 months for the first 2 years and then semiannually. In patients with an increase in PSA level above the nadir, novel imaging consisting of PSMA-11 PET and/or CT and bone scan was performed. The ethics committee of the Medical University of Vienna approved the study (permit 1460). All patients provided written informed consent prior to SLND. The data are presented according to the STARD criteria [19].

### Imaging protocol

PET/CT and PET/MRI were performed as previously described [12]. Briefly, [^68^Ga]-PSMA-11 at 2 MBq per kg body weight was injected intravenously 60 min before the PET/MRI(CT) acquisition was started. PET/MRI was performed on a Biograph mMR (Siemens, Germany), consisting of an MRI-compatible PET detector integrated with a 3.0-T whole-body MRI scanner. The PET component used a three-dimensional acquisition technique and offered an axial field of view (FOV) of approximately 23 cm and a transverse FOV of 45 cm with a sensitivity of 13.2 cps/kBq. For improved image quality, forced diuresis was induced with 20 mg of furosemide administered intravenously before PSMA, and all patients undergoing PET/MRI received a bladder catheter.

Local PET of the pelvis included a 10-min list-mode acquisition starting 60 min after injection. Partial body PET (skull base to thighs) was performed with four bed positions, each with a 4-min sinogram mode. Reconstruction parameters for PET were: three iterations per 21 subsets, and summation of the 10-min pelvic acquisition for visual and semiquantitative analysis. MRI-based attenuation correction was applied using DIXON VIBE sequences comprising in-phase and opposed-phase, and fat-saturated and water-saturated images.

PET/CT was performed from the vertex to the upper thighs using a 64-row multidetector hybrid system (Biograph TruePoint 64; Siemens, Erlangen, Germany), with an axial FOV of 216 mm, a PET sensitivity of 7.6 cps/kBq and a transaxial PET resolution of 4–5 mm (full-width at half-maximum). PET acquisition parameters were 4 min per bed position, four iterations per 21 subsets, 5-mm slice thickness, and 168 × 168 matrix, using the point-spread-function-based reconstruction algorithm TrueX. CT maps were used for PET attenuation correction.

The MRI protocol included native axial T2w turbo spin echo, coronal T1w spin echo and axial diffusion-weighted imaging sequences of the pelvis, and T2w HASTE and DIXON VIBE partial body imaging. The MRI parameters have been described in detail previously [12]. PET/MRI was performed in 42 patients.

Venous-phase contrast-enhanced CT was performed after intravenous injection of 100 ml of a tri-iodinated, non-ionic contrast medium at a rate of 2 ml/s with tube voltage 120 mA, tube current 230 kV, collimation 64 × 0.6 mm, 3-mm slice thickness at a 2-mm increment, and 512 × 512 matrix. PET/CT was performed in 23 patients.

### Image analysis

**Fig. 1:**
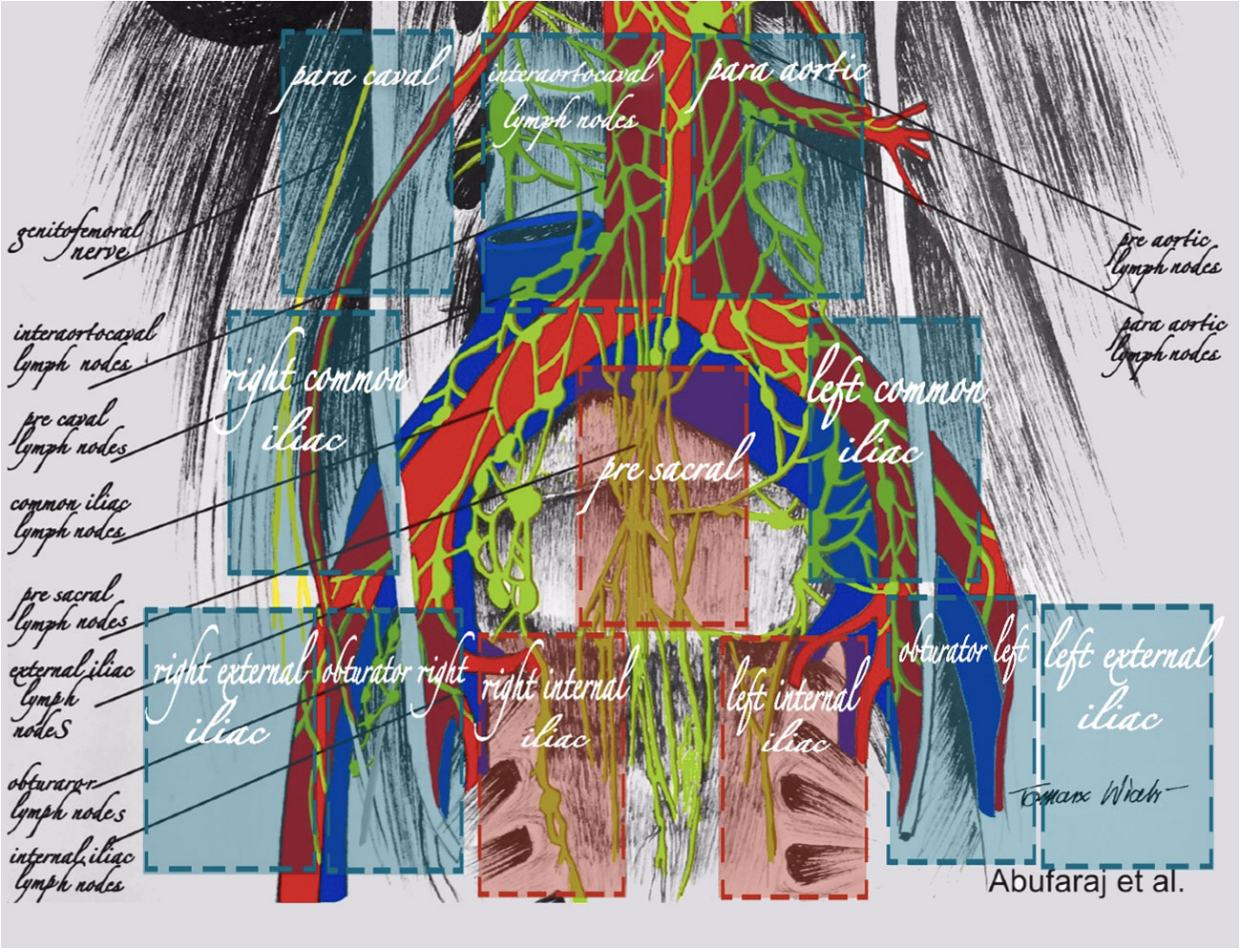
Schematic tomographic image of the templates for patients undergoing complete bilateral extended salvage lymph node dissection

PET/MRI and PET/CT images were reviewed separately by two experienced certified nuclear medicine physicians using a Hermes Hybrid 3D workstation (Hermes Medical Solutions, Stockholm) for PET/MRI and PET/CT. MR/CT images were assessed according to section 3 of RECIST 1.1 (Measurability of Tumors at Baseline) using AGFA IMPAXX EE software for MRI and CT [20]. Every focus showing differentially increased uptake compared to the surrounding background uptake in a visible morphological structure (e.g. LN) was considered a positive finding. Foci showing slight increases in uptake of ^68^Ga-PSMA in the area of the paravertebral sympathetic ganglia were ignored, as they are known to be false-positive [21]. Documentation was carried out concerning LNs according to the scheme shown in Fig. 1.

### Salvage lymph node dissection

All patients underwent full bilateral superextended pelvic LN dissection up to the level of the inferior mesenteric artery. The SLND templates included all the LNs from the following major regions: right and left pelvis, and presacral and retroperitoneal regions. For the subregional analysis, the pelvic templates included nine subregions: bilateral common iliac, external iliac, obturator and internal iliac regions and the presacral region. The retroperitoneal templates comprised three subregions: periaortic, interaortocaval and precaval LNs between the aortic bifurcation and the inferior mesenteric artery. The external, internal and obturator subregions were evaluated as a group to minimize localization/attribution errors. All SLND were performed by the same surgeon (S.F.S.) according to a standard prespecified surgical protocol, and the LNs were sent separately to histopathology according to the prespecified templates.

### Histopathological methods and analysis

Formalin-fixed LN specimens were completely embedded in paraffin and processed according to the protocol described by Rais-Bahrami et al. [22]. Paraffin-embedded sections of thickness 2 µm were routinely stained with haematoxylin and eosin, and where required immunohistochemical staining with a pancytokeratin antibody (AE1/3, dilution 1:100) was performed to exclude micrometastases.

### Statistical analysis

This was a prospective study designed to test the performance of SLND in patients with pelvic-only LN metastases on PSMA-11 PET after RP. We report the data of the first 65 patients who met our predefined inclusion criteria. A descriptive analysis of the whole cohort was performed. Diagnostic performance from the regional and subregional analyses was compared with histopathology after SLND as the confirmatory test. A confirmed LN metastasis in a region or subregion was considered positive for the template analysis. The sensitivity, specificity, positive predictive value (PPV) and negative predictive value (NPV) with corresponding confidence intervals (CI) of PSMA-11 PET/CT(MRI) were estimated for the assessment of LN metastasis per region and subregion using contingency tables. Statistical associations were tested using the chi-squared test or Fisher’s exact test as appropriate. Statistical analyses were performed using STATA 13 (Stata Corp., College Station, TX, USA) with an alpha level of 0.05 indicating statistical significance. Accuracy was calculated using MedCalc Software (Ostend, Belgium) according to the following formula: $=\frac{\boldsymbol{TP}+\boldsymbol{TN}}{\boldsymbol{TP}+\boldsymbol{TN}+\boldsymbol{FP}+\boldsymbol{FN}}$, where TP is true positive, TN is true negative, FP is false positive and FN is false negative. The images were reviewed prior to SLND. The pathology and PSMA-11 PET/CT(MRI) were each interpreted blinded to the result of the other.

## Results

### Baseline characteristics of patients

Overall, 65 consecutive patients meeting the inclusion criteria were included in the study. Table 1 summarizes patient and tumour characteristics at the time of RP and SLND. About three-quarters of patients showed extraprostatic extension on their RP specimens and one-fifth initially harboured LN metastases at the time of RP. Before SLND, 50 patients (76.9%) had additional therapy after RP with 17 (26.2%) receiving ADT and 33 (50.8%) radiotherapy. Intraoperative and postoperative complications were reported in 7 patients (10.8%) and 21 patients (32.3%), respectively. The detailed oncological and perioperative and postoperative outcomes, including complications of SLND and postoperative complications, will be reported separately as a part of an ongoing clinical trial.

**Table 1:** Baseline characteristics of 65 patients with prostate cancer who underwent salvage lymph node dissection for biochemical recurrence after radical prostatectomy

Characteristic	Value
Age at RP (years), median (IQR)	61 (59–66)
PSA level prior to RP (ng/ml), median (IQR)	9 (7–12)
Pathological stage at RP, *n* (%)	
pT2	12 (18.5)
pT3a	30 (46.6)
pT3b	20 (30.8)
pT4	3 (4.6)
Lymph node stage at RP, *n* (%)	
pN0	57 (87.7)
pN1	8 (12.3)
Number of lymph nodes removed at RP, median (IQR)	8 (5–12)
Positive surgical margin, *n* (%)	23 (35.4)
Time to BCR (months), median (IQR)	12 (2–23)
Therapy after RP, *n* (%)	
Hormonal	17 (26.2)
Radiation	33 (50.8)
Pathological Gleason score, *n* (%)	
<7	1 (1.5)
7 (3 + 4)	14 (21.5)
7 (4 + 3)	20 (30.8)
>7	30 (46.2)
Age at SLND (years), median (IQR)	65 (63–69)
PSA level at SLND (ng/ml), median (IQR)	1.4 (0.8–2.9)
Number of lymph nodes removed, median (IQR)	40 (33–48)
Number of positive lymph nodes removed, median (IQR)	4 (2–5)
Time from SLND to last follow-up (months), median (IQR)	13 (7–22)
BCR after SLND, *n* (%)	35 (61.4)
Operative time (minutes), median (IQR)	210 (180–230)
Length of hospital stay (days), median (IQR)	6 (6–8)

*RP* radical prostatectomy, *SLND* salvage lymph node dissection, *IQR* interquartile range, *BCR* biochemical recurrence, *PSA* prostate-specific antigen

### Findings of PSMA-11 PET/CT(MRI) and SLND

**Fig. 2:**
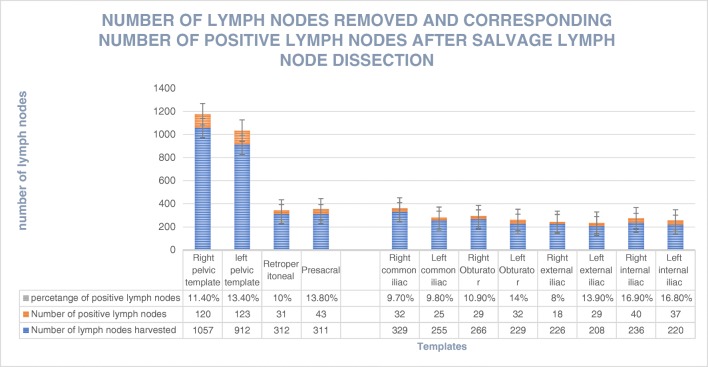
Numbers of lymph nodes removed and corresponding numbers of positive lymph nodes after salvage lymph node dissection

On PSMA-11 PET/CT(MRI), all but two patients (97%) had positive LNs in the pelvic region only. Two patients had retroperitoneal LN involvement in addition to pelvic LNs. Figure 2 shows the numbers of harvested LN in each region and subregion, the corresponding numbers of positive LN, and the rates of positivity. Metastases were detected in 13.8% of LNs resected on SLND (317 of 2,292). The largest number of resected LNs was from the right common iliac subregion (329) followed by the retroperitoneal region (312). The rate of positive LNs across all subregions ranged from 8% to 16.9%. The largest percentage of positive LNs was in the right internal iliac subregion (16.9%) followed by the left internal iliac region (16.8%). The median diameter of LNs positive on PSMA-11 PET/CT(MRI) was 7.2 mm (IQR 5.3–9 mm), whereas the median diameter of false-negative LNs was 3.4 mm (IQR 2.1–5.4 mm; *p* = 0.01).

### Regional and subregional analyses with corresponding histopathology after SLND

The sensitivity, specificity, PPV, NPV and diagnostic accuracy in the regional and subregional analyses are presented in Table 2. Overall, the diagnostic accuracy for all the subregions ranged from 88% to 99%. The accuracies in detecting LN metastases in the right and left pelvic regions were 95% (95% CI 87.1–99%) and 99% (95% CI 91.7–100%), respectively, and in the presacral region and retroperitoneal regions were both 95% (95% CI 87.1–99%). The accuracy in detecting LN metastases in the common and external iliac LNs ranged from 88% to 97%, and the accuracy in the internal iliac and obturator regions was >95%. As a test for predicting the probability of the presence of LN metastasis, the PPVs were higher than 92% and reached 100%. The NPVs ranged from 86% to 98%. Grouping external, internal and obturator LNs together, both the PPV and NPV in detecting LN metastases ranged from 97% to 100% in all regions (the grouping was done to account for possible attribution bias in location assignment on imaging versus surgery).

**Table 2:** Sensitivity, specificity, positive and negative predictive values, and diagnostic accuracy of PSMA-11 PET/CT(MRI) in 65 patients from the regional and subregional analyses

	Sensitivity (95% CI), %	Specificity (95% CI), %	Positive predictive value (95% CI), %	Negative predictive value (95% CI), %	Diagnostic accuracy (95% CI), %
Regional analysis					
Pelvic (right)	94.6 (81.8–99.3)	96.4 (81.7–9.9)	97.2 (83.6–99.6)	93.1 (77.8–98.1)	95.4 (87.1–99)
Pelvic (left)	100 (90.5–100)	96.4 (81.7–99.9)	97.4 (84.4–99.6)	100	98.5 (91.7–100)
Presacral	90.9 (70.8–98.9)	97.7 (87.7–99.9)	95.2 (74.2–99.3)	95.5 (84.8–98.8)	95.4 (87.1–99)
Retroperitoneal	72.7 (39–939)	100 (93.4–100)	100	94.7 (87.3–97.9)	95.4 (87.1–99)
Subregional analysis					
Common iliac					
Right	65 (40.8–84.6)	97.8 (88.2–99.9)	92.9 (64.6–98.9)	86.3 (77.6–92)	87.7 (77.2–94.5)
Left	78.6 (49.2–95.3)	98 (89.6–100)	91.7 (60.8–98.7)	94.3 (85.9–97.9)	93.9 (85–98.3)
External iliac					
Right	80 (51.9–95.7)	100 (92.3–100)	100	94.3 (85.8–97.9)	95.4 (87.1–99)
Left	92.9 (66.1–99.8)	98 (89.6–100)	92.9 (65–98.9)	98 (88.3–99.7)	96.9 (89.3–99.6)
Internal iliac					
Right	95 (75.1–99.9)	100 (92–100)	100	97.8 (86.7–99.7)	98.4 (91.6–100)
Left	87 (66.4–97.2)	100 (91.6–100)	100	93.3 (83–97.6)	95.4 (87.1–99)
Obturator					
Right	89.5 (66.9–98.7)	100 (92.3–100)	100	95.8 (86.1–98.8)	96.9 (89.3–99.6)
Left	84.2 (60.4–96.6)	100 (92.3–100)	100	93.9 (84.4–97.7)	95.4 (87.1–99)
External, internal and obturator combined					
Right	97.1 (84.7–99.9)	100 (88.8–100)	100	96.9 (81.8–99.5)	98.5 (91.7–100)
Left	100 (90.3–100)	96.6 (82.2–99.9)	97.3 (84–99.6)	100	98.5 (91.7–100)

*p* < 0.001, Fisher’s exact test among all regions and subregions

### Subgroup analysis

The performance of PSMA-11 PET/CT(MRI) was evaluated in relation to PSA level at the time of SLND (Table 3). The PPV in patients with a PSA level ≥1.4 ng/ml (the median PSA level in our cohort) was almost always 100% in all regions and subregions except the presacral region, where it was 93% (95% CI 67.5–99). In patients with a PSA level <1.4 ng/ml, the PPV in the regional analysis ranged from 94% to 100%, and in the subregional analysis, ranged from 50% in the right common iliac LNs to 100% in the external and internal iliac and obturator LNs. No statistically significant differences in PSMA-11 PET performance in relation to primary Gleason score and time to BCR were found. Furthermore, no differences in imaging performance were found between PSMA-11 PET/CT (in 23 patients) and PSMA-11 PET/MRI (in 42 patients).

**Table 3:** Positive and negative predictive values for PSMA-11 PET/CT(MRI) in 65 patients in relation to PSA level at the time of imaging

	PSA level at the time of SLND (ng/ml)^a^			
	<1.4		≥1.4	
	Positive predictive value (95% CI), %	Negative predictive value (95% CI), %	Positive predictive value (95% CI), %	Negative predictive value (95% CI), %
Regional analysis				
Pelvic (right)	95 (74.2–99.2)	100	100	85.7 (61.7–95.7)
Pelvic (left)	94.4 (71.8–99.1)	100	100	100
Presacral	100	97 (84.2–99.9)	93.3 (67.5–99)	94.1 (70.6–99)
Retroperitoneal	100	96.7 (84.2–99.4)	100	92.6 (79.5–97.6)
Subregional analysis				
Common iliac				
Right	50 (7.1–92.9)	90.3 (84.1–94.3)	100	80 (63.1–90.3)
Left	80 (34.5–96.8)	89.3 (77.9–95.2)	100	100
External iliac				
Right	100	96.2 (80–99.4)	100	92.6 (79.5–97.6)
Left	85.7 (46.7–97.6)	100	100	96 (79.3–99.3)
Internal iliac				
Right	100	96.3 (80.9–99.4)	100	100
Left	100	90.9 (73.7–97.3)	100	95.7 (77.4–99.3)
Obturator				
Right	100	96.2 (80–99.4)	100	95.5 (76.4–99.3)
Left	100	96.3 (80.9–99.4)	100	90.9 (73.8–97.3)
External, internal and obturator combined				
Right	100	100	100	93.3 (67.6–99)
Left	95 (74.2–9.2)	100	100	100

^a^33 patients with a PSA level <1.4 ng/ml, median 0.8 ng/ml (IQR 0.6–1 ng/ml); 32 patients with a PSA level ≥1.4 ng/ml, median 2.9 ng/ml (IQR 2.3–4.2 ng/ml)

## Discussion

In this prospective study, PSMA-11 PET/CT(MRI) showed a high diagnostic accuracy for nodal relapse after RP according to topographical location using histopathological results as the confirmatory test. All men were included in a consecutive manner without any obvious selection bias. All underwent a complete bilateral template-based SLND for assumed LN-only relapse based on PSMA-11 PET imaging. The high performance, as illustrated by the high PPV, of PSMA-11 PET/CT(MRI) was confirmed by regional and subregional analyses. The diagnostic accuracy of PSMA-11 PET/CT(MRI) increased with the size of LN metastatic deposits and PSA levels. Nevertheless, its performance was still good in patients with small metastatic deposits and in those with low PSA levels. The data show that PSMA-11 PET/CT(MRI) detects LN metastases with high PPV and NPV. This suggests a possible role of site-directed surgical intervention in patients with LN-only metastasis after RP, reinforcing the promise of personalized medicine in PC.

Conventional morphological imaging techniques such as CT and MRI have suboptimal diagnostic performance in detecting LN metastases with sensitivities ranging from 13% to 40% [23]. Despite the ability of PET/CT with choline analogues to detect LNs missed by conventional cross-sectional imaging, its performance in identifying single LN metastases or the extent of nodal relapse is poor. This is mainly due to the limited spatial resolution of most PET scanners (5 mm) and the different biochemical mechanism (choline as a metabolic marker rather than PSMA-11 as a receptor-binding ligand) [14]. In agreement with our data, the detection of recurrence and its location has been found to depend largely on LN size and PSA level [14]. In contrast to the findings of previous studies, the primary Gleason score and the time to BCR were not associated with PSMA-11 PET performance in this study. This might be explained by our highly selected cohort of patients with BCR, who were included in the study and underwent SLND only if LN-only recurrence was considered to be present with no evidence of further distant PSMA-avid lesions. Furthermore, almost 80% of the patients in our cohort had high-risk or unfavourable intermediate-risk disease based on the histopathological specimen from RP, and only a few patients had low-risk disease (1 patient) and favourable intermediate-risk disease (14 patients). This might also have limited the difference in PSMA-11 PET performance in relation to Gleason score. Moreover, the reported lesion-based pooled sensitivity was limited [24]. Current literature indicates that the performance of PSMA PET is higher in patients with higher PSA levels [11]. In line with this finding, PSMA PET showed higher diagnostic performance in the group of patients with PSA levels higher than the median (1.4 ng/ml). Nevertheless, the accuracy of PSMA PET was still high in patients with PSA levels <1.4 ng/ml.

PSMA-11 PET showed promising potential in identifying the sites of BCR because of the high-affinity binding of PSMA to folate hydrolase of PC cells [25], and is gaining worldwide popularity as a clinically relevant staging tool, even in men with low PSA levels (i.e. <1 ng/ml) at recurrence [26]. Indeed, positive PSMA-11 PET/CT scans have been reported in 44% and 79% of patients with PSA levels ≤1 and 1–2 ng/ml, respectively [27].

Among all patients with metastatic PC, those with LN relapse limited to the regional and/or retroperitoneal LNs may be a distinct group with favourable outcomes if treated adequately. Indeed, long-term complete biochemical remission has been reported in patients who underwent PET/CT-guided surgical resection of low-volume nodal metastases [28]. Moreover, there is a growing body of data indicating a beneficial impact of surgical resection on survival in these patients [7]. In this study, approximately four out of ten patients remained disease-free during follow-up. This is in line with short-term outcomes found in previous studies on SLND in patients with LN-only recurrence based on choline PET or PSMA-11 PET [11, 28]. Nevertheless, long-term data on SLND based on PSMA-11 PET are still limited. Furthermore, a first randomized phase 3 prospective trial designed to determine whether PSMA-11 PET/CT molecular imaging can improve outcomes in patients with early BCR following RP is currently recruiting, highlighting the increasing role of PSMA-11 PET imaging in the tailoring of salvage therapies for BCR after RP [29]. In addition, accurate assessment of the presence of isolated LN relapse and identifying the precise site of positive LN metastases is important for site-directed surgery. Indeed, we have already implemented a radioguided surgical approach to reduce potential risks, costs and morbidity of complete bilateral extended SLND as done in our current study [30].

The main limitation of our study was the attribution of dissected LNs to relative regions and subregions. This is known to lead to systematic operator-dependent nomenclature errors that would potentially have affected the results and limited their reproducibility. The PPV in the subregion analysis ranging from 50% to 100% is likely to have resulted from attribution bias from imaging versus surgery. Incomplete removal of involved LNs during surgery also has to be taken into account, as the PPV of PSMA-11 PET is known to be very high. Our study population included a very selected group of patients with LN-only recurrence. By excluding patients with bone or visceral metastases, selection bias was, indeed, one of the limitations of this study. About one-quarter of our cohort underwent ADT prior to imaging, which might have affected the rate of PSMA expression. However, in this relatively small study, subgroup analysis did not show a difference in PSMA-11 PET performance between patients with and without previous ADT (data not shown). In addition, we also do not have data about the extent or template of LN dissection at the time of RP. However, we reported the number of LN harvested, which was lower than the number found on SLND. Moreover, the number of LN harvested is in agreement with the trend in contemporary RP series in which the number of SLND procedures performed and the number of LN harvested are generally decreasing. On the other hand, this study included, to our knowledge, the largest prospective consecutive cohort and evaluated the accuracy of PSMA-11 PET/CT(MRI) in patients undergoing SLND with bilateral extended templates. Besides, the regional and subregional analyses in this study provide evidence of the benefit of implementing site-directed surgery, as the NPV was very high. In this study, the number of LNs harvested during SLND was relatively high, reflecting the completeness of template dissection. Higher LN yield is known to be associated with a higher likelihood of complications such as symptomatic lymphocele. The rate of metastases in these LNs was in accordance with previously reported rates for PSMA-11 PET [31]. Nevertheless, this rate was lower than that reported for PET using choline derivatives (32%) [10].

### Conclusion

In this prospective study, PSMA-11 PET/CT(MRI) had high diagnostic accuracy in detecting and localizing LN metastases in men with BCR after RP. The high correlation between PET/CT(MRI) and surgical findings provides evidence of the benefit of site-directed therapeutic surgical intervention that would avoid the possible morbidities of full bilateral extended SLND in a subset of patients.
